# Physiochemical and Thermal Characterization of Municipal Solid Waste and Agricultural Residue Blends for Torrefaction

**DOI:** 10.1155/tswj/3310369

**Published:** 2026-04-21

**Authors:** Ibrahim Luqman Mpungu, Josphat Igadwa Mwasiagi, Benson Dulo, Obadiah Maube, Patrick Nziu, Ocident Bongomin

**Affiliations:** ^1^ Department of Manufacturing, Textile and Industrial Engineering, School of Engineering, Moi University, P.O. Box 3900, Eldoret, Kenya, mu.ac.ke; ^2^ Department of Technical Teacher and Instructor Education, School of Education, Kyambogo University, P.O. Box 1, Kyambogo, Uganda, kyu.ac.ug; ^3^ African Centre of Excellence II in Phytochemical, Textile, and Renewable Energy (ACE II PTRE), Moi University, P.O. Box 3900-30100, Eldoret, Kenya, mu.ac.ke; ^4^ Department of Industrial and Mechatronics Engineering, School of Mechanical and Manufacturing Engineering, Technical University of Kenya, P.O. Box 52428-00200, Nairobi, Kenya, tukenya.ac.ke; ^5^ Department of Mechanical Engineering, Walter Sisulu University, P.O. Box 1421 East London 5200, Mthatha, South Africa, wsu.ac.za

**Keywords:** characterization, coffee husk, corn cobs, municipal solid waste, torrefaction

## Abstract

Municipal solid waste (MSW), together with other biomass resources, presents a viable feedstock for renewable energy production; however, its direct conversion is limited by high moisture content, ash content, and heterogeneous composition. These challenges can be mitigated through torrefaction, provided that suitable feedstock selection and optimization are achieved. This study investigates the physicochemical and thermal characteristics of MSW, coffee husks (CH), corn cobs (CC), and their blends to demonstrate how biomass quality can be improved through blending. MSW was blended with CH or CC at mass ratios of 25:75, 50:50, and 75:25 (db/db%). Proximate, ultimate, lignocellulosic, thermogravimetric, and calorific value analyses were conducted. Proximate analysis showed that MSW had the highest moisture (10.102 ± 0.141%), volatile matter (71.115 ± 0.759%), and ash content (6.674 ± 0.477%), whereas CH exhibited the highest fixed carbon content (18.863 ± 0.572%). Ultimate analysis revealed that MSW contained the highest hydrogen content (6.911 ± 0.183%), CH had the highest carbon content (50.001 ± 0.184%), and CC showed the highest oxygen (44.185 ± 0.273%), nitrogen (1.395 ± 0.045%), and sulfur (0.057 ± 0.035%) contents. MSW had the lowest hemicellulose (11.941 ± 0.269%) and cellulose (19.334 ± 0.294%) contents, while CC had the lowest lignin content (12.304 ± 0.219%). The calorific value of MSW (17.01 ± 0.292 MJ kg^−1^) increased upon blending, reaching up to 17.59 ± 0.241 MJ kg^−1^. Thermogravimetric analysis indicated enhanced thermal degradation rates with increasing MSW content in the blends. In conclusion, blending MSW with agricultural residues significantly improves its physicochemical and thermal properties, enhancing its suitability for torrefaction. The 25MSW75CH blend demonstrated the most favorable characteristics and is recommended as an optimal feedstock for torrefaction‐based waste‐to‐energy applications at industrial scale.

## 1. Introduction

Global energy demand is projected to increase by approximately 28% between 2015 and 2040, driven by rapid population growth, technological advancement, and industrial expansion [1, 2]. At present, this demand is predominantly met by fossil fuels, which are increasingly unsustainable, costly, and associated with severe environmental and public health impacts, including greenhouse gas emissions and air pollution [3]. These challenges necessitate a transition toward renewable energy sources that are affordable, widely available, and environmentally sustainable [4]. In this context, waste‐to‐energy (WtE) technologies have gained growing attention as viable pathways for simultaneous waste management and energy recovery [5]. Recent advances in WtE have expanded beyond conventional incineration to include advanced thermochemical and hybrid conversion routes such as torrefaction‐assisted co‐firing, integrated gasification systems, and thermal upgrading strategies aimed at improving fuel quality, process efficiency, and emissions performance [6, 7]. Emerging studies highlight that pretreatment processes, including torrefaction and co‐processing, can significantly enhance biomass homogeneity, grindability, and energy density, thereby improving compatibility with existing energy infrastructure and reducing operational challenges [8, 9]. However, the sustainability and effectiveness of these advanced WtE pathways are strongly dependent on appropriate feedstock selection and optimization [4]. Biomass, defined as organic material derived from plant and animal sources, can be obtained from woody and nonwoody plants, agricultural and forest residues, agro‐industrial by‐products, and municipal solid waste (MSW), offering a diverse and underutilized resource base for sustainable energy generation [6, 7].

Among various biomass resources, MSW is particularly attractive for energy generation due to its widespread availability, potential cost‐effectiveness, and versatility for conversion into multiple fuel forms [4]. MSW is generated continuously from residential, commercial, institutional, and industrial activities and consists of a complex mixture of organic and inorganic fractions, including food residues, garden waste, paper, plastics, textiles, wood, and minor quantities of metals and glass [4, 6, 10]. Annually, global MSW generation is estimated at 1.30–2.01 billion tonnes and is projected to rise sharply to approximately 3.40–4.20 billion tonnes by 2050, driven by population growth, rapid urbanization, economic development, and changing consumption patterns [1, 3, 11–14]. Importantly, MSW composition varies significantly with regional economic status, cultural practices, climate, and waste management policies, influencing both the proportion of biodegradable organic matter and the prevalence of noncombustible or high‐ash components [15]. In low‐ and middle‐income regions, MSW is typically dominated by biodegradable organic fractions, whereas higher‐income regions generate waste streams with increased plastics, paper, and packaging materials. Modern consumer lifestyles have further increased compositional heterogeneity through the growing presence of synthetic materials, multilayer packaging, and moisture‐rich food waste, complicating thermal conversion and fuel standardization [3]. This heterogeneity directly affects fuel properties such as moisture content, ash yield, calorific value, and devolatilization behavior, thereby posing challenges for efficient energy recovery and necessitating appropriate pretreatment, segregation, or blending strategies prior to thermochemical conversion [5, 12, 14].

To mitigate the environmental and health risks associated with unmanaged MSW, its utilization in renewable energy production has gained increasing attention [11, 16]. This approach not only reduces greenhouse gas emissions and public health burdens but also contributes to energy security and circular economy objectives. Studies by Amulen et al. [11] and Abdulyekeen et al. [6] further demonstrate that effective MSW valorization supports multiple United Nations Sustainable Development Goals (SDG3, SDG6, SDG7, SDG11, and SDG13). Despite its substantial untapped energy potential, direct thermal conversion of MSW remains challenging due to its high moisture content, low calorific value, fibrous nature, and pronounced compositional heterogeneity [16, 17]. To overcome these constraints, torrefaction pretreatment has been widely proposed as a viable upgrading strategy [5, 6]. In parallel, co‐combustion strategies have been investigated, wherein MSW or its refuse‐derived fuel (RDF) fraction is co‐fired with conventional solid fuels such as coal, lignite, or biomass to stabilize combustion, improve thermal efficiency, and reduce fossil fuel consumption [18, 19]. For instance, recent studies have demonstrated successful MSW combustion and co‐combustion with lignite, highlighting improved burnout characteristics and reduced emissions when appropriate blending ratios are employed [20].

Torrefaction is a mild thermochemical pretreatment process typically carried out at temperatures between 200°C and 300°C under an inert or oxygen‐limited atmosphere. During torrefaction, biomass undergoes partial devolatilization and dehydration, resulting in the breakdown of hemicellulose, reduction of moisture content, and enrichment of fixed carbon. These transformations improve key fuel properties, including higher heating value (HHV), energy density, hydrophobicity, grindability, and storage stability, while also producing a more homogeneous solid fuel suitable for downstream thermal conversion processes such as combustion, gasification, and pyrolysis [21–23].

MSW is a highly heterogeneous material composed of fractions that interact during thermal conversion rather than degrading independently [16]. When MSW is blended with more compositionally uniform agricultural biomass, synergistic effects can arise due to complementary physicochemical properties of the individual components. Agricultural residues typically exhibit higher fixed carbon, lignocellulosic content, and thermal stability, whereas MSW contains higher volatile matter, ash, and inorganic species. During thermal treatment, these contrasting characteristics promote interactions such as improved heat transfer, moderated devolatilization, and altered reaction pathways, resulting in enhanced carbonization efficiency and more stable solid products. Liang et al. [24] reported that blending MSW with biomass can normalize elemental composition and reduce variability in fuel quality. Similarly, Zheng, Lee, and Lin [25] demonstrated that co‐processing MSW with agricultural wastes enhances fixed carbon yield and thermal stability during carbonization, thereby improving suitability for torrefaction and downstream energy applications. These synergistic interactions underpin the rationale for blending MSW with agricultural residues as a strategy to upgrade low‐quality waste feedstocks into more uniform and energy‐dense solid fuels.

Agricultural residues globally contribute over 1 billion tons of biomass, with top crops like sugarcane, maize, rice, and wheat generating around 2 billion tons of waste [13, 24]. This study will utilize corn cobs (CC) and coffee husks (CH), major waste generators in Uganda’s maize and coffee value chains [26]. Previous research on biomass blending, such as Zheng, Lee, and Lin’s [25] co‐processing of textile sludge and lignocellulose biowaste, shows increased heating value, grindability, and hydrophobicity while the ash content is reduced. Other studies, like those by Zheng, Lee, Lin, et al. [25], Manatura et al. [27], and Lin and Zheng [28], further confirm that co‐torrefaction with blending improves biochar quality, enhances heating value, and ensures high energy yields.

Although extensive research has examined biomass blending and co‐torrefaction performance, most studies focus on homogeneous agricultural or industrial residues, with limited attention given to the fundamental characterization of MSW blended with agricultural biomass prior to torrefaction. In particular, the physicochemical and thermal interactions between MSW and regionally abundant agricultural residues remain insufficiently understood. This study addresses this gap by systematically characterizing MSW, CC, CH, and their blends using proximate, ultimate, lignocellulosic, calorific, and thermogravimetric analyses (TGAs). By elucidating how blending modifies fuel properties, thermal degradation behavior, and energy potential, this work provides critical insights into feedstock selection and blend optimization for torrefaction‐based WtE systems. The findings contribute to improved process design and operational efficiency, particularly for developing‐country contexts where MSW and agricultural residues coexist as underutilized energy resources.

## 2. Materials and Methods

### 2.1. Sample Collection and Preparation

In this study, MSW was collected from the Kitezi landfill site in Kampala (0° 25′ 0″ N and 32° 34′ 00″ W), Uganda. From a previous study, the MSW composition is 37.8% food waste, 33.6% yard waste, 6.7% paper, 0.8% metals, 7.8% plastics, 8.6% stones and debris, 1.3% textiles, 0.7% glasses, and 2.7% miscellaneous [29]. The nonrecyclable portion of MSW, comprising food and yard waste, was considered for this study. Agricultural wastes, specifically 50 kg of CH and 50 kg of CC, were collected from Grainpulse Limited, an agribusiness company located in Mukono District, Uganda.

To remove excess moisture from the feedstock, the samples were oven‐dried at 105°C for 24 h following ASTM E871‐82 (2019) standard procedures for moisture determination in biomass [30]. The dried biomass was then size‐reduced by grinding and sieving through a 35‐mesh screen (0.50 mm opening) to ensure particle size uniformity. Subsequently, MSW was blended with either CH or CC at mass ratios of 0:100, 25:75, 50:50, 75:25, and 100:0 (db/db%), in accordance with established biomass blending protocols [28], as summarized in Table 1. The prepared samples were finally sealed in airtight plastic bags and stored at room temperature prior to further characterization and analysis.

**TABLE 1:** Sample nomenclature.

Sample	Blend ratio	Pseudo‐name/code
Individual	100% municipal solid wastes	MSW
	100% coffee husks	CH
	100% corn cobs	CC

Blend of municipal solid wastes with coffee husks	25% municipal solid wastes and 75% coffee husks	25MSW75CH
	50% municipal solid wastes and 50% coffee husks	50MSW50CH
	75% municipal solid wastes and 25% coffee husks	75MSW25CH

Blend of municipal solid wastes with corn cobs	25% municipal solid wastes and 75% corn cobs	25MSW75CC
	50% municipal solid wastes and 50% corn cobs	50MSW50CC
	75% municipal solid wastes and 25% corn cobs	75MSW25CC

### 2.2. Characterization

#### 2.2.1. Proximate Analysis

The proximate properties of the feedstocks were determined using a macrothermogravimetric analyzer (ELTRA THERMOSTEP Thermogravimetric Analyzer, ELTRA GmbH, Germany) in accordance with ASTM E1131‐08 [31]. The instrument is equipped with an integrated high‐precision digital balance and is operated under computer control using TGA software (Version 1.4.2.12). Homogenized samples were placed in ceramic crucibles and subjected to programmed heating under controlled nonoxidizing and oxidizing atmospheres. Moisture content, volatile matter, and fixed carbon were determined under a nonoxidizing environment, while ash content was measured under an oxidizing atmosphere, following the thermal decomposition and mass‐loss profiles defined by the ASTM E1131‐08 protocol.

#### 2.2.2. Lignocellulose Composition

The samples’ lignocellulosic composition was analyzed via Neutral Detergent Fibre (NDF), Acid Detergent Fibre (ADF), and Acid Detergent Lignin (ADL), according to the Van Soest method [32]. The ADL represented the biomass’s lignin content. The cellulose content was given by the difference between the ADF and ADL, whereas the hemicellulose content was given by the difference between the NDF and the ADF.

#### 2.2.3. Ultimate Analysis

The ultimate analysis was conducted to determine the elemental composition of the samples. Carbon (C), hydrogen (H), nitrogen (N), and sulfur (S) contents were measured using a CHNS automatic elemental analyzer (Elementar Vario EL III, Germany) in accordance with ASTM D5373. The analysis was performed under controlled combustion conditions as specified by the standard. Oxygen (O) content was calculated by difference, following ASTM D3176, by subtracting the measured percentages of C, H, N, S, ash, and moisture from 100 wt.% on a dry basis [25].

#### 2.2.4. Calorific Value

The HHV of the samples was determined using an automated digital bomb calorimeter (IKA C2000 calorimeter) in accordance with ASTM D5865. Approximately 700 mg of each dried and homogenized sample was placed in the combustion crucible and subjected to complete combustion under oxygen in a sealed bomb within an adiabatic environment. The calorimeter employs integrated software, an internal heater, and a circulating water system to ensure thermal stabilization during combustion. The HHV was calculated automatically from the measured temperature rise of the adiabatic system and displayed digitally. All analyses described in Sections 2.2.1–2.2.4 were performed in triplicate, and the reported values represent the mean of the measurements.

#### 2.2.5. TGA

TGA and derivative thermogravimetric (DTG) analysis were conducted using the same macrothermogravimetric analyzer (ELTRA THERMOSTEP Thermogravimetric Analyzer), following the ASTM E1131‐08 standard. Approximately 20–30 mg of each dried and homogenized biomass sample was placed in an alumina crucible and heated under an inert nitrogen atmosphere (purity ≥ 99.99%). The temperature was increased from ambient temperature to 900°C at a constant heating rate of 10°C min^−1^, with a nitrogen flow rate of 50 mL min^−1^ to ensure nonoxidative conditions. The mass loss of the sample was continuously recorded as a function of temperature and time. The DTG curves were obtained by differentiating the TGA weight‐loss data to identify distinct thermal degradation stages corresponding to moisture evaporation, devolatilization, and char decomposition. This approach enables detailed assessment of the thermal behavior and degradation kinetics of MSW, CH, CC, and their blends [33].

## 3. Results and Discussion

### 3.1. Proximate, Ultimate, and Lignocellulosic Composition

#### 3.1.1. Proximate Analysis

The proximate analysis results of the samples are presented in Table 2. The moisture content of MSW, CH, and CC was 10.722 ± 0.141%, 6.522 ± 0.127%, and 9.558 ± 0.075%, respectively. These values are in range with those recorded by Chhabra et al. [34] and Triyono et al. [35] for MSW (9.0%–10.92%); Tadesse et al. [36], Mukherjee et al. [37], and Bongomin et al. [31] for CH (2.7%–9.323%); and Ibitoye et al. [38] and Klaas et al. [39] for CC (8.7%–11.0%). The moisture content of the blends decreased with a percentage increase in the CH or CC composition in the blends. The decrease in moisture content of MSW could be due to the lignocellulosic composition of both CH and CC that were used for blending. However, the moisture content of the blends of MSW with CC was higher than that of MSW with CH, partly, since both individual wastes have higher moisture content than CH, and it is woodier than CC.

**TABLE 2:** Proximate analysis of MSW, CH, CC, and their blends.

Biomass sample	Moisture (%)	Volatile matter (%)	Fixed carbon (%)	Ash (%)
MSW	10.102 ± 0.141	71.115 ± 0.759	13.018 ± 0.359	6.674 ± 0.477
CH	6.522 ± 0.127	68.265 ± 0.942	18.863 ± 0.572	5.307 ± 0.407
CC	9.558 ± 0.075	70.827 ± 0.741	12.504 ± 1.082	6.173 ± 0.461
25MSW75CH	6.021 ± 0.143	68.762 ± 0.136	17.394 ± 1.248	5.827 ± 0.197
50MSW50CH	6.924 ± 0.094	69.281 ± 0.235	16.235 ± 0.420	6.011 ± 0.115
75MSW25CH	7.172 ± 0.354	70.135 ± 1.850	15.829 ± 1.564	6.212 ± 0.015
25MSW75CC	8.021 ± 0.027	72.469 ± 2.012	14.117 ± 1.755	6.118 ± 1.326
50MSW50CC	8.852 ± 0.107	71.004 ± 0.241	13.814 ± 0.129	6.130 ± 0.185
75MSW25CC	9.053 ± 0.180	70.101 ± 0.259	13.248 ± 0.255	6.418 ± 0.211
MSW^1^	10.790 ± 0.000	76.970 ± 0.000	15.940 ± 0.000	7.080 ± 0.000
CH^1^	9.323 ± 0.000	77.789 ± 0.000	15.301 ± 0.000	3.910 ± 0.000
CC^1^	8.700 ± 0.000	71.120 ± 0.000	10.600 ± 0.000	10.120 ± 0.000

*Note:* MSW^1^ [35] CH^1^ [36] CC^1^ [38].

Negi et al. [40] compared the effect of moisture content of biomass on fuel quality. They found that high moisture content affects the fuel’s HHV. Fuels with low moisture content often possess high flame temperatures, quickly complete combustion, and have short residence times in the combustion chamber and are therefore recommended [36]. From the results in Table 2, although all blends do not have worrying moisture content percentages, the 25MSW75CH blend has the least moisture content and thus is the most desired.

Volatile matter contains hydrogen, oxygen, and light hydrocarbons [41]. The volatile matter of MSW, CH, and CC was 71.115 ± 0.759%, 68.265 ± 0.942%, and 70.827 ± 0.741%, respectively, as shown in Table 2. The values obtained are within range with those recorded by Chhabra et al. [34] and Triyono et al. [35] for MSW (52.80%–76.97%); Tadesse et al. [36] and Bongomin et al. [31] for CH (62.90%–77.789%); and Ibitoye et al. [38] and Klaas et al. [39] for CC (70.00%–71.12%). It is clear that the volatile matter decreased with CH addition in the blended sample, while it increased with CC addition. The high volatile matter content in the blends of MSW with CC could be influenced by the high moisture content of the individual biomasses. A high percentage of volatile matter leads to fast vaporization of most biomass, and thus volatiles have a strong impact on the thermal degradation and combustion behavior of biomass [36]. Although high volatile matter in biomass indicates ease of biomass ignition during combustion, fuels with high volatile matter often emit a lot of smoke [42]. From Table 2, the sample 25MSW75CC has the highest volatile matter. Biomass containing a high percentage of volatile matter burns faster, and this affects the biochar yield when torrefied.

In Table 2, the fixed carbon of MSW, CH, and CC is 13.138 ± 0.359%, 18.863 ± 0.572%, and 12.504 ± 1.082%, respectively. The values obtained are in close range with those noted by Chhabra et al. [34] and Triyono et al. [35] for MSW (8.00%–15.94%); Tadesse et al. [36] and Bongomin et al. [31] for CH (15.301%–18.37%); and Ibitoye et al. [38] and Klaas et al. [39] for CC (9.20%–10.60%). It was noted that CH has the highest fixed carbon, while CC had the lowest percentage. However, the fixed carbon of MSW increased with an increase in both CH and CC percentages in the blended sample. The increase in the fixed carbon composition in blends of MSW with CC could be caused by the high carbon content in both MSW and CC. The presence of high quantities of fixed carbon in biomass signifies its suitability for energy conversion. Therefore, the 25MSW75CH blend would be the most desired sample since it has the highest fixed carbon. Furthermore, a high fixed carbon content is needed in order for a fuel to release more heat during combustion and thus corresponds to an increase in the mass yield and HHV of a fuel [39, 41].

Ash is an undesirable component of biomass. Fuels with higher than 6% ash content often affect gasification and combustion systems [43]. Ash forms slag that leads to fouling and agglomeration in boilers and gasifier systems. Table 2 shows the ash content of MSW, CH, and CC as 6.674 ± 0.477%, 5.307 ± 0.407%, and 6.173 ± 0.461%, respectively. Apart from CH, whose ash content was in range (3.91%–10.06%) as recorded by Tadesse et al. [36] and Bongomin et al. [31], the ash content of other samples are not in range with that noted by Chhabra et al. [34] and Triyono et al. [35] for MSW (7.08%–39.20%) and Ibitoye et al. [38] and Klaas et al. [39] for CC (9.80%–10.12%). The deviation could be attributed to the variation in the MSW composition or the type of maize from which the CC was obtained.

The ash content of MSW decreased with an increase in either CH or CC percentage in the blended samples. However, the ash content of the blended samples was greater than that of the CH and CC. This could be due to the increase in the inorganic composition due to dirt in the blended sample by the addition of MSW. In a study by Awoyale et al. [44] on the elemental composition of ash from different biomass, they disclosed that blending of biomass affects the elemental composition of the ash. Ash is an undesired component of biomass if it is to be used as an energy source, and blending can be done to reduce its content [25]. From Table 2, only the 25MSW75CH blend has an ash content of less than 6%, making it the best blend for energy production in terms of proximate analysis.

Ordinarily, the quality of biomass can be improved through torrefaction [6]. The torrefaction process can reduce the original moisture content of a fuel by 98% [45]. This could be due to the significant release of bound and unbound water in the biomass when heated at a temperature range of 100°C–200°C [45]. Furthermore, the moisture content can also be reduced due to devolatilization and carbonization of hemicellulose and cellulose in the biomass. During the torrefaction process, the decrease in moisture content depends on the process conditions [6]. Ibitoye et al. [38] noted that the moisture content of corn cobs decreased from 8.7% to 1.56% when the process conditions were 200°C for 20 min, while it decreased to 0.98% when process parameters were 240°C for 40 min.

On the same note, decreasing the moisture content of biomass results in a decrease in the volatile matter, while the fixed carbon and ash content increase. However, researchers such as Lau et al. [41] and Stępień and Bialowiec [46] caution that increasing the torrefaction conditions, specifically temperature and residence time, can negatively impact the mass yield of the biochar. Biomass with high moisture content is often characterized by a low mass yield [40]. In their study on torrefaction of empty fruit bunches, Sukiran et al. [45] found that a decrease in the moisture content of the feedstock translated to a significant increase in the yield of the biochar. The increase in mass yield was attributed to the evaporation of water in the feedstock during the torrefaction process.

Torrefaction also decreases the volatile matter of biomass at rates proportional to the torrefaction process parameters and chemical nature of the biomass [45, 47]. The volatile matter of biochar decreases because of dehydration and the decarboxylation reaction [25]. Decreasing the volatile matter of biomass decreases its burnout rate. This was experienced by Tadesse et al. [36] when the volatile matter of torrefied biomass decreased to 50.45% from a range of 77.789%–78.437% for raw biomass. However, the decrease in burnout rate was more prominent when torrefaction was done at higher temperatures, as the volatile matter of biomass is further degraded, the liquid yield from the biomass increases, resulting in biomass mass loss [41, 48].

The ash content increases after torrefaction due to the accumulation of a high concentration of inorganic substances as a result of mass loss [49]. Note that the percentage quantity of ash increases with the severity of the torrefaction process parameters [49, 50]. However, biomass with high ash content can produce high biochar yields at low/mild torrefaction temperatures [50]. In their study, Nakason et al. [50] concluded that the high ash content makes the degradation of biomass difficult, thus decreasing biomass’s devolatilization rate.

Many authors have recently noted that blending can be used to enhance the quality of biomass before torrefaction. Viegas et al. [51] mixed microalgae (*Chlorella vulgaris*) and lignocellulosic biomass and noticed that while the volatile matter and ash content decreased with the increase of lignocellulose composition in the sample, the fixed carbon and moisture content increased. Zheng et al. [52] studied the impact of blending textile sludge with lignocellulose biowaste and noticed an increase in the volatile matter and fixed carbon contents of the biochar. The changes in the volatile matter and fixed carbon compositions of the blended waste samples are often influenced by the lignocellulose composition of the waste [52]. Zheng et al. [52] recorded high ash content reductions after torrefying blended biomass. However, they emphasize the impact of choosing suitable biomass when blending such that the biochar properties can be enhanced.

#### 3.1.2. Ultimate Analysis

Table 3 shows the ultimate analysis of the samples. Although various elements are typically present in biomass, the main elements normally include carbon, hydrogen, and oxygen, which are vital in constructing hemicellulose, cellulose, and lignin [32]. Also, nitrogen and sulfur often occur in small percentages, which are sometimes undetectable or negligible [32, 36]. The carbon content of MSW, CH, and CC was 48.050 ± 0.263%, 50.001 ± 0.184%, and 47.050 ± 0.284%, respectively. Whereas the carbon content was in range with values recorded by Tadesse et al. [36] and Bongomin et al. [31] for CH (47.38%–50.001%) and Ibitoye et al. [38] and Klaas et al. [39], and Laohalidanond and Kerdsuwan [53] for CC (36.40%–47.04%), the MSW values recorded were out of range when compared to 43.2%–44.72% [10, 54, 55]. This disparity in the carbon content of MSW could have been due to the difference in the composition of MSW samples used in the different studies.

**TABLE 3:** Ultimate analysis of MSW, CH, CC, and their blends.

Biomass sample	Carbon (%)	Hydrogen (%)	Oxygen^∗^ (%)	Nitrogen (%)	Sulfur (%)
MSW	48.050 ± 0.263	6.911 ± 0.183	44.162 ± 0.216	0.856 ± 0.006	0.021 ± 0.013
CH	50.001 ± 0.184	6.325 ± 0.269	42.703 ± 0.271	0.959 ± 0.011	0.012 ± 0.008
CC	47.050 ± 0.284	6.183 ± 0.185	44.185 ± 0.273	1.395 ± 0.045	0.057 ± 0.035
25MSW75CH	50.052 ± 0.248	6.140 ± 0.215	42.746 ± 0.231	0.898 ± 0.015	0.001 ± 0.001
50MSW50CH	49.037 ± 0.217	6.259 ± 0.184	43.830 ± 0.251	0.873 ± 0.021	0.006 ± 0.002
75MSW25CH	48.385 ± 0.195	6.328 ± 0.156	44.557 ± 0.252	0.868 ± 0.014	0.020 ± 0.012
25MSW75CC	49.067 ± 0.267	6.579 ± 0.163	44.223 ± 0.183	1.121 ± 0.042	0.031 ± 0.021
50MSW50CC	48.281 ± 0.261	6.619 ± 0.169	44.892 ± 0.321	1.001 ± 0.023	0.027 ± 0.016
75MSW25CC	48.185 ± 0.216	6.682 ± 0.342	44.201 ± 0.233	1.096 ± 0.024	0.020 ± 0.005
MSW^1^	43.200 ± 0.900	8.100 ± 0.160	47.900 ± 0.900	0.780 ± 0.010	0.030 ± 0.000
CH^1^	44.418 ± 0.000	5.787 ± 0.000	49.795 ± 0.000	BDL ± 0.000	BDL ± 0.000
CC^1^	41.24 ± 0.000	6.10 ± 0.000	35.87 ± 0.000	0.12 ± 0.000	1.56 ± 0.000

*Note:* MSW^1^ [35] CH^1^ [36] CC^1^ [38].

Abbreviation: BDL, below detection limit.

^∗^Results obtained by difference (O = 100–[C + H + N + S + ASH]).

The carbon content of MSW was increased with an increase in both the CH and CC composition in the blended samples, even though the carbon content of CC was lower than that of MSW. This could have occurred since blending MSW with CC altered the lignocellulosic composition of the sample, thereby influencing the carbon content of biomass. Carbon is the most desired component in biomass being used for fuel generation [56]. High carbon content depicts a high calorific value of biomass [57, 58] and a decrease in the COx emissions in the environment [59].

In Table 3, the hydrogen content of MSW, CH, and CC was 6.911 ± 0.183%, 6.325 ± 0.269%, and 6.183 ± 0.185%, respectively. These values are in range with those noted by Ivanovski et al. [60], Rahman et al. [54], and Siritheerasas et al. [61] for MSW (5.67%–8.1%); Tadesse et al. [36] and Bongomin et al. [31] for CH (6.325%–6.566%); and Ibitoye et al. [38] and Klaas et al. [39] for CC (6.10%–6.20%). The hydrogen content of MSW decreased with an increase in the blend percentage of either CH or CC. The decrease in hydrogen could be attributed to the decrease in the moisture content of the blended samples. Hydrogen bonds that retain additional water in biomass are formed due to the presence of hydroxyl (‐OH) groups [62]. Although hydrogen is an important source of heat generation in the combustion process, high hydrogen content is usually accompanied by low carbon content [63].

Oxygen is required during thermal degradation of the volatile components in biomass [64]. The oxygen content of MSW, CH, and CC was 44.162 ± 0.216%, 42.703 ± 0.271%, and 44.185 ± 0.273%, respectively, in Table 3. These values are in range with those noted by Ivanovski et al. [60], Rahman et al. [54], and Siritheerasas et al. [61] for MSW (44.26%–49.83%); Tadesse et al. [36] and Bongomin et al. [31] for CH (32.581%–46.052%); and Ibitoye et al. [38] and Klaas et al. [39] for CC (35.87%–47.1%). Whereas blending with high percentages of CH resulted in a decrease in the oxygen content of MSW, blending with low CH percentages or CC resulted in an increase in the oxygen content of MSW. Blending with high CH and CC percentages results in significant reductions in the moisture content of the samples as compared with blend samples with low CH and CC percentages. However, Nazos et al. [65] alluded to the fact that reduction of moisture and other gases affects the oxygen content of biomass. Decreasing the oxygen content in biomass enhances the characteristics of the resulting biofuel since oxygen negatively contributes to the HHV of any fuel [59].

The nitrogen and sulfur content in all the biomass samples was low compared to those of conventional fuels. This might result in a reduction in NOx and SOx emissions when biomass is burnt in the atmosphere [66]. Whereas the nitrogen content of MSW, CH, and CC was 0.856 ± 0.006%, 0.959 ± 0.011%, and 1.395 ± 0.045%, respectively, the sulfur content of MSW, CH, and CC was 0.021 ± 0.013%, 0.012 ± 0.008%, and 0.057 ± 0.035%, respectively. These nitrogen content values recorded are within range with those noted by Ivanovski et al. [60], Rahman et al. [54], and Siritheerasas et al. [61] for MSW (0.78%–3.49%); Tadesse et al. [36] and Bongomin et al. [31] for CH (below detection limit [BDL]–0.959%); and Ibitoye et al. [38] and Klaas et al. [39] for CC (0.12%–0.5%). Equally, the sulfur content values are in range with those noted by Ivanovski et al. [60], Rahman et al. [54], and Siritheerasas et al. [61] for MSW (0.03%–0.40%); Tadesse et al. [36] and Bongomin et al. [31] for CH (BDL–0.072%); and Ibitoye et al. [38] and Klaas et al. [39] for CC (< 0.05%–1.56%). These values are so low that they are insufficient to emit sulfur and nitrogen oxides during combustion, which makes the biomass blends environmentally secure fuels [28]. The nitrogen content of CC was out of range compared to results from previous studies, probably due to the soil nutrients used during cultivation, as was noted by Limo et al. [67]. On the other hand, the sulfur content of MSW could be out of range due to the difference in the MSW composition used in the various studies.

Typically, torrefaction improves the characteristics of biomass by increasing the carbon content while the hydrogen, oxygen, and sulfur contents decrease [10, 68]. Although not desired in energy conversions, the nitrogen content also increases with torrefaction [57]. The increase in the carbon content during torrefaction is due to the degradation of hemicellulose [68]. This is because torrefaction occurs at lower temperatures, leaving lignin, which has the highest carbon content, almost unaltered. This might explain why the carbon content of biomass seems to increase with torrefaction temperature since more lignin will be decomposed with increasing torrefaction temperatures [57].

Ivanovski et al. [60] and Samad et al. [69] emphasize that the carbon content increases while the hydrogen and oxygen content decrease during torrefaction owing to the release of water vapor and formation of CO and CO2 as the volatile matter escapes. Decreases in H/C and O/C atomic ratios of torrefied blended biomass have been recorded by other researchers. In a study by Zheng et al. [52], textile sludge was blended with different biowaste and later torrefied. Although the H/C and O/C atomic ratios decreased with torrefaction of the textile sludge and the individual biowastes, further decreases in the H/C and O/C atomic ratios were recorded for the blends of textile sludge and each biowaste after torrefaction. Elsewhere, Huang et al. [70] blended sewage sludge with leucaena. It was noticed that although pure leucaena had relatively low atomic H/C and O/C ratios, the atomic ratios further decreased with an increase in the blend ratio of sewage sludge during torrefaction. As demonstrated by Guo et al. [64] and Zheng et al. [52], the lower the H/C and O/C atomic ratios, the higher the fuel quality. Thus, overall, the 25MSW75CH blend is recommended for torrefaction because of its high carbon content and low H/C and O/C atomic ratios.

#### 3.1.3. Lignocellulose Composition

Typically, the main components of biomass include hemicellulose, cellulose, and lignin. The proportions of these constituents depend on the type and geographical origin of biomass, among other factors [71, 72]. Chen et al. [32] ranks the percentage composition of each constituent as cellulose > hemicellulose > lignin.

In Table 4, the hemicellulose, cellulose, and lignin compositions of MSW, CH, and CC were 11.941 ± 0.269%, 19.334 ± 0.294%, and 16.343 ± 0.219%; 23.883 ± 0.285%, 30.726 ± 0.163%, and 20.775 ± 0.285%; and 31.049 ± 0.284%, 32.755 ± 0.195%, and 12.304 ± 0.219%, respectively. From other researchers, the composition of MSW is in the range of hemicellulose (7.20%–16.50%), cellulose (15.30%–65.80%), and lignin (11.40%–43.80%) [6, 73]. Similarly, the CH composition is in range with the hemicellulose (21.9%–46.3%), cellulose (33.7%–41.6%), and lignin (15.6%–27.56%) composition declared by Tadesse et al. [36], Khan et al. [74], and Obafemi et al. [75]. The variation in the cellulose composition of CH can be influenced by the kind of CH used in the study. Yiga et al. [76] recorded that the cellulose composition of untreated CH was 42.1% and 25.4% for Arabica and Robusta coffee, respectively. Gerassimidou et al. [77] also noted that the composition of lignin differs not only between plant species but also between parts of the same plant. The CC compositions is in range with the hemicellulose (25%–35%), cellulose (32.3%–55%), and lignin (6.7%–30%) composition recorded by Olupot et al. [26], Lee et al. [78], and Nayak and Mukherjee [79].

**TABLE 4:** Lignocellulose composition of MSW, CH, CC, and their blends.

Biomass sample	Hemicellulose (%)	Cellulose (%)	Lignin (%)
MSW	11.941 ± 0.269	19.334 ± 0.294	16.343 ± 0.219
CH	23.883 ± 0.285	30.726 ± 0.163	20.775 ± 0.285
CC	31.049 ± 0.284	32.755 ± 0.195	12.304 ± 0.219
25MSW75 CH	17.461 ± 0.175	24.726 ± 0.292	18.314 ± 0.261
50MSW50CH	16.981 ± 0.296	20.785 ± 0.273	16.814 ± 0.179
75MSW25CH	16.941 ± 0.275	19.804 ± 0.169	16.143 ± 0.261
25MSW75CC	18.246 ± 0.213	25.716 ± 0.241	14.215 ± 0.198
50MSW50CC	17.040 ± 0.251	21.794 ± 0.154	13.925 ± 0.278
75MSW25CC	16.853 ± 0.259	20.812 ± 0.195	13.025 ± 0.275
MSW^1^	15.450 ± 0.070	27.800 ± 0.100	17.700 ± 0.050
CH^1^	21.900 ± 0.000	41.600 ± 0.000	27.560 ± 0.000
CC^1^	35.000 ± 0.000	45.000 ± 0.000	15.000 ± 0.000

*Note:* MSW^1^ [73] CH^1^ [36] CC^1^ [79].

The hemicellulose, cellulose, and lignin composition of MSW increased with the addition of either CH or CC. The percentage increase of the lignocellulose composition depended on the CH or CC percentage in the blended sample. Biomass with a high lignin content often produces very little ash when combusted and burns for longer times [76]. Khan et al. [74] noted that lignin does not have a well‐defined primary structure and its composition tends to vary from sample to sample. Lignin works as a binding element for cellulose and hemicellulose structures [80].

During biomass energy conversion, the lignocellulose composition of biomass has a great impact on its energy yields. Whereas Chen et al. [32] noted that of all the lignocellulose components that respond to higher energy content, cellulose plays a major role due to its higher carbon content, Araújo et al. [68] and Menya et al. [66], on the other hand, note that the lignin content contributes to higher carbon content, thus a higher energy yield as compared to the cellulose content. Negi et al. [40] noted that it is much more difficult to hydrate lignin and hence convert it to char than both hemicellulose and cellulose. Furthermore, Chen and Kuo [81] stated that during severe torrefaction, lignin plays a more important role in energy contribution than cellulose since a huge percentage of cellulose is consumed in the process. Thus, all three lignocellulose components contribute to energy generation.

During the torrefaction process, lignocellulosic compositions of biomass are affected differently. While the hemicellulose and cellulose percentages decrease, the lignin percentage increases depending on the torrefaction temperature and holding time [65]. In a study to torrefy cocoa pod husk, Surono et al. [82] noticed a decrease in the hemicellulose content from 28.99% to 3.68% and the cellulose content from 13.14% to 1.43% due to an increase in the torrefaction temperature from 200°C to 300°C for 60 min. They also observed that torrefying the same sample at a constant temperature (250°C) while increasing the holding time from 0 to 90 min resulted in a decrease in the hemicellulose content from 28.99% to 7.95%. The lignin content increased from 28.99% to 72.4% as the torrefaction temperature increased from 200°C to 300°C for 60 min and rose from 33.07% to 58.57% at 250°C when the holding time was increased to 90 min. The increment was not due to a lignin increase in the sample but rather a decrease in other components, especially hemicellulose. Nazos et al. [65] also asserted that at moderate torrefaction process parameters, low molecular weight lignin accumulates on the material surface.

During torrefaction, degradation of the different constituents of biomass occurs at different temperatures [82]. Hemicellulose is known to degrade at 100°C–320°C, cellulose breaks down between 300°C and 400°C, and lignin decomposes between 200°C and 800°C [6, 65]. This results in the degradation of more hemicellulose quantities than cellulose quantities [32, 33]. This is because hemicellulose has a poor level of polymerization, resulting in low resistance to thermal, mechanical, and chemical degradation [83]. However, the rate of decomposition of cellulose increases when reaction temperature increases above 300°C [33].

Lignin is known to be hydrophobic as opposed to both hemicellulose and cellulose and is highly resistant to chemical and biological degradation [72]. The hydrophobicity of hemicellulose is low, medium for cellulose, and high for lignin [40]. The holocellulose (hemicellulose and cellulose) adsorbs moisture because of the free hydroxyl groups possessed, which easily attract water and hold water molecules through the hydrogen bond [82]. The decrease in the holocellulose after torrefaction translates into the hydrophobic nature of torrefied biomass. This is because torrefaction leads to the loss of hydroxyl groups of the holocellulose [82]. Hydrophobicity in biomass enhances its resistance to rotting and fungal attack, which improves the control of fuel storage conditions, characteristics associated with good fuels [84].

Biomass loses weight after torrefaction due to moisture and light volatile matter removal [85] accompanied by thermal cracking and devolatilization of the biomass lignocellulosic structure [58]. In a study by Nazos et al. [65], after torrefaction, agricultural feedstock mass yield was lower compared to that of woody biomass because the former had more hemicellulose composition. A similar occurrence was noticed by Sarker et al. [58], who also attributed the reduction of mass yield to the high hemicellulose content in agricultural biomass, which degrades faster than other biomass constituents. Therefore, to reduce the mass loss that is synonymous with torrefaction [84], biomass with low hemicellulose composition is better suited for torrefaction.

### 3.2. Calorific Value

**FIGURE 1:**
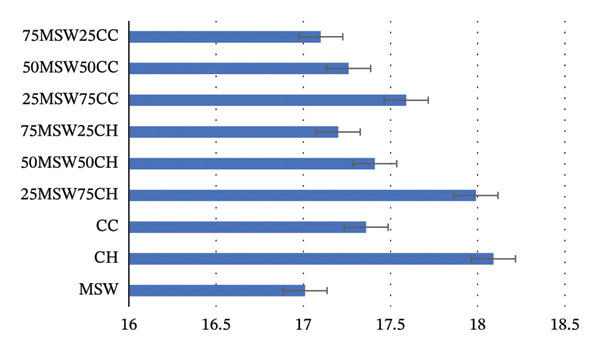
The calorific value of MSW, CH, CC, and their blends.

The calorific value is used to describe the energy potential of biomass [86]. It also helps in determining the thermal conversion efficiency of any biomass material [26]. The calorific value of MSW, CH, and CC was 17.01 ± 0.292 MJ/kg, 18.09 ± 0.139 MJ/kg, and 17.36 ± 0.293 MJ/kg, respectively. These values are within limits of those noted by Ivanovski et al. [60], Rahman et al. [54], and Siritheerasas et al. [61] for MSW (16.42–24.3 MJ/kg); Tadesse et al. [36] and Bongomin et al. [31] for CH (18.26–20.68 MJ/kg); and Ibitoye et al. [38], Klaas et al. [39], and Obafemi et al. [75] for CC (12.6–17 MJ/kg). The effect of blending MSW with CH and CC is given in Figure 1. From Figure 1, it is evident that the calorific value of MSW increased with an increase in either CH or CC percentage in the blend sample. These values could be influenced by the moisture content of the biomass. The calorific value of biomass is associated with its moisture content and depends on the chemical composition of biomass [87]. For MSW, the calorific value is also influenced by biowaste composition, as demonstrated by Tumuluru et al. [88] in their study to briquette MSW bales with variable moisture content. It was noted that the higher calorific value possessed by MSW compared to that of herbaceous biomasses was due to the high (30%) lignin content and also the plastic materials in the MSW bales.

Ibitoye et al. [38] and Thabuot et al. [86] asserted that the calorific value is influenced by the quantity of fixed carbon in biomass. However, the order of the fixed carbon percentage (CH > MSW > CC) is contrary to the order of the calorific value percentage (CH > CC > MSW) in our study. Also, the order of the calorific value percentage is also contrary to the order of the lignin composition percentage (CH > MSW > CC), although Lunguleasa et al. [89] opined that the possession of high lignin content in biomass translates into a high calorific value. This deviation might be due to the high ash and moisture content possessed by the MSW, which is known to negatively impact the calorific value of biomass [90]. Likewise, Chen et al. [32] noted that fuels with high ash and moisture content have lower calorific values, which corresponds to our results.

Torrefaction increases the calorific value of biomass [65]. During torrefaction, the increase in the calorific values is proportional to the torrefaction temperatures and residence time [68, 87]. However, the mass yield is decreased in the process, which affects the biochar yield [85, 91]. In another study, Dirgantara et al. [92] stated that when the residence time is increased during torrefaction, the oxygen and hydrogen in biomass form water vapor, carbon dioxide, and carbon monoxide that are released to the atmosphere. This decreases the O/C and H/C ratios that result in high calorific values [87]. Lunguleasa et al. [91] further illustrated that whereas the calorific value of pellets increased from 17.87 to 18.682 MJ/kg when the temperature increased from 200°C to 300°C with a residence time of 3 min, the calorific value changed from 18.157 to 19.225 MJ/kg and from 19.120 to 21.291 MJ/kg when the residence times were 5 and 10 min, respectively. They further indicated that the mass loss was 8.6%, 13.3%, and 20.2% for 3, 5, and 10 min, respectively.

Different kinds of biomass can be blended during torrefaction to further enhance the calorific value of the biochar. This was illustrated by Zheng et al. [52], who observed that the calorific value and energy density of the biochar increased with an increase in the blending ratio of textile sludge with other lignocellulose biowaste. Torrefied textile sludge had a calorific value of 18.7 MJ/kg compared to 19.7 MJ/kg possessed by the blend of textile sludge with macadamia husk (30/70% db). It was noticed that macadamia husk had the highest carbon content and percentage of fixed carbon compared to the rest of the lignocellulose biowaste. Therefore, biomass with high carbon content, fixed carbon, and lignin composition is better suited when co‐torrefying due to the parameters’ relevance to energy conversion. Chen et al. [32], in their study on the principles, applications, and challenges of biomass torrefaction, confirmed the positive correlation that exists between the lignin content and quality of the torrefied product. They recorded higher calorific values for lignin (23.3–26.6 MJ/kg) compared to cellulose and hemicellulose (approximately between 17 and 18 MJ/kg).

Whereas torrefaction is known to improve the calorific value of biomass, Zheng et al. [52] noted that co‐torrefaction due to blending of textile sludge with agricultural and lignocellulose biowaste further enhanced the calorific value of biomass. The calorific value further increased with an increase in the blending ratio of biowaste [28]. Therefore, in this study, the 25MSW75CH blend would be recommended for torrefaction because of its high calorific value.

### 3.3. TGA

The TGA is commonly used to study the thermal decomposition of biomass [15]. TGA analysis of MSW, CH, and CC was conducted, and the results are presented in Figure 2(a). From Figure 2(a), the weight loss of the biomass samples as the temperature increases from room temperature to 1000°C is shown. From 28°C to approximately 104°C, a slight and constant biomass weight loss was observed. This initial weight loss was attributed to the evaporation of moisture in the biomass. Compared to Goli et al.’s [93] finding that weight loss during this stage is often < 10%, in our study, CH, MSW, and CC lost < 3%, < 5%, and < 8%, respectively. It should be noted that weight loss in this case is mainly due to the loss of moisture content in the particular biomass.

FIGURE 2TGA (a) and DTG (b) curves for MSW, CH, and CC.

**Figure:**
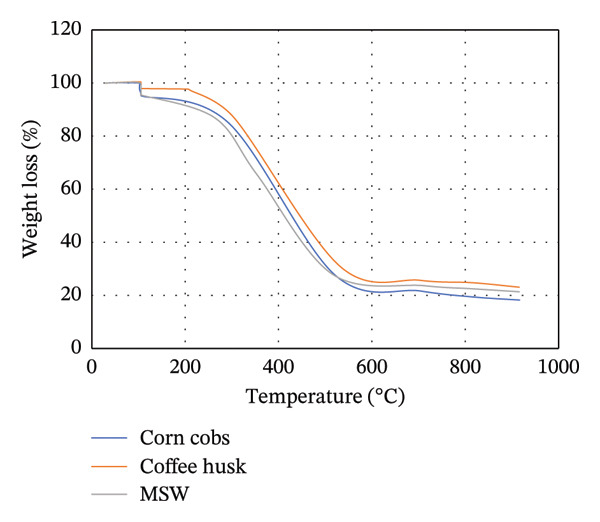
(a)

**Figure:**
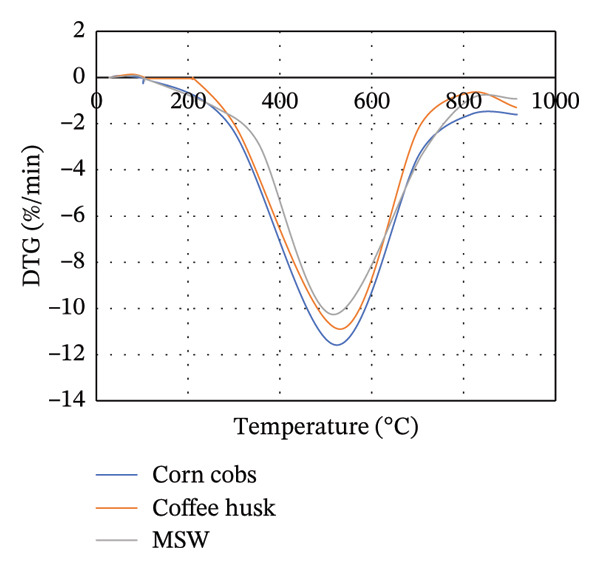
(b)

Beyond 104°C, the weight slightly drops to approximately 250°C before it drastically decomposes. It is within these temperature ranges that thermal decomposition of the chemical components in the biomass begins. Biomass mainly comprises hemicellulose, cellulose, and lignin, which degrade within temperature ranges of 199°C–327°C, 226°C–366°C, and 287°C–800°C, respectively [68, 94]. The fast degradation of MSW could be due to its high volatile content (71.115 ± 0.759%) compared to that of CH (68.265 ± 0.942%) and CC (70.827 ± 0.741%).

Unlike hemicellulose, cellulose has a slower decomposition reaction at temperatures below 250°C, and the thermal decomposition rate of biomass increases when the reaction temperature is increased above 300°C [33]. Pang et al. [95] further noted that cellulose also possesses the narrowest decomposition range. Lignin is difficult to decompose due to its complex chemical composition [63], and thus its thermal degradation occurs slowly over an extensive temperature range from 200°C to 800°C [95]. For CC and CH, lignocellulose thermal degradation is pronounced in ranges of 300°C–530°C, while it is in ranges of 260°C–530°C for MSW. Lignin is responsible for the major portion of the char product, and its decomposition beyond 800°C leaves behind residues comprising ash, tar, and fixed carbon [96].

In Figure 2(b), the DTG curves show the two degradation stages in the thermal decomposition of the biomass during combustion. A major decomposition rate is observed at about 105°C for all biomass, followed by additional weight degradation with time. At 105°C, the decrease in weight per unit time was more pronounced in MSW, while the least decrease was recorded for CH. This could be attributed to the high moisture content possessed by MSW (10.722 ± 0.141%) compared to CC (9.558 ± 0.075%) and CH (6.522 ± 0.127%).

Lubwama et al. [96] noted that DTG thermograms illustrate the decomposition maximums in single peaks due to the degradation of cellulose, and the point of highest intensity corresponds to the peak temperature at which the respective decomposition dominantly occurs. From Figure 2(b), it is evident that the biomasses’ peak temperatures ranged between approximately 515.39°C and 520.88°C, with MSW and CC having the lowest and highest peaks, respectively. Decomposition of biomass continues after the peak temperatures up to approximately 900°C. There is minimal weight change with time between approximately 900°C and 915°C due to the formation of char.

### 3.4. Effect of Blending on the Thermal Degradation of Waste

To illustrate the thermal decomposition characteristics of the blends of MSW with either CH or CC at different ratios, TGA was done, and the results are displayed in Figure 3. For all the blends, the weight loss can be divided into three stages: evaporation of moisture content and devolatilization of light volatile matter, extensive thermal degradation of organic matter, and limited devolatilization, as was described by Lin and Zheng [28] and Zheng et al. [52]. However, the temperatures at which each of these stages occurs vary depending on the blend composition and ratio.

FIGURE 3TGA and DTG curves for the (a) 25MSW75CH, (b) 50MSW50CH, (c) 75MSW25CH, (d) 25MSW75CC, (e) 50MSW50CC, and (f) 75MSW25CC.

**Figure:**
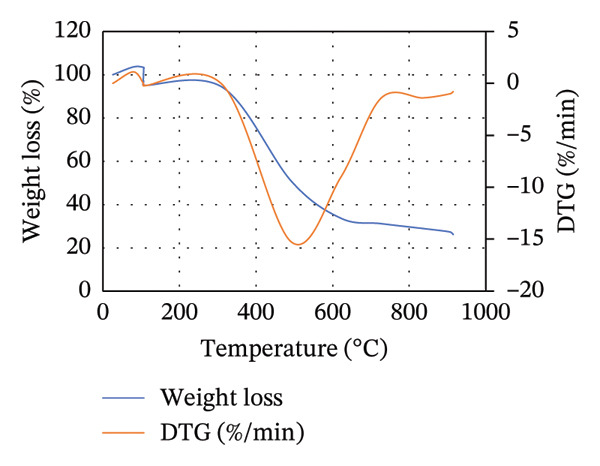
(a)

**Figure:**
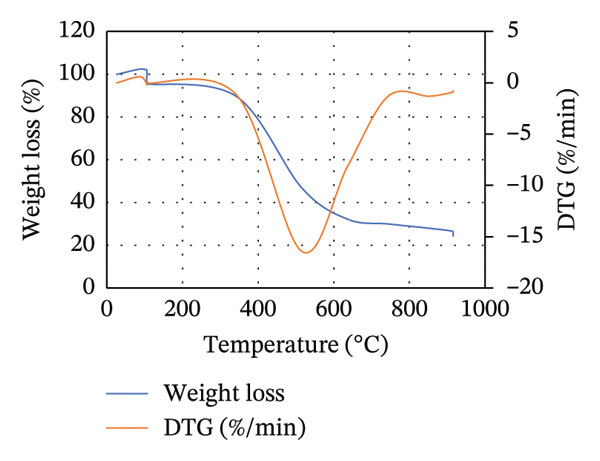
(b)

**Figure:**
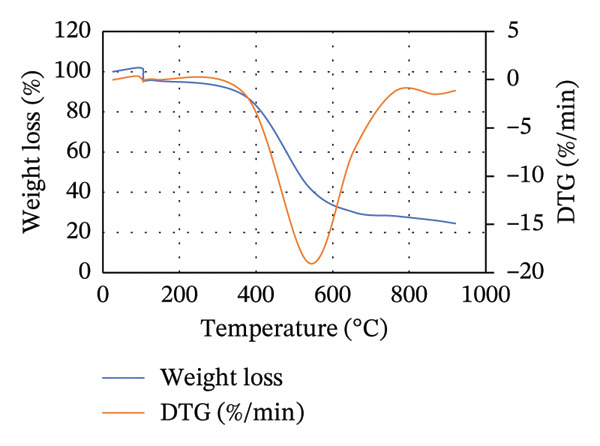
(c)

**Figure:**
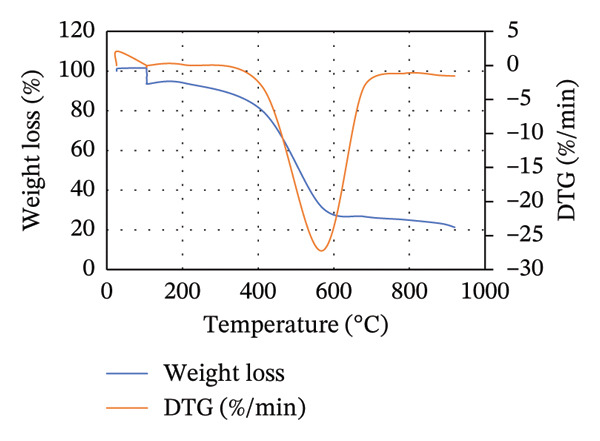
(d)

**Figure:**
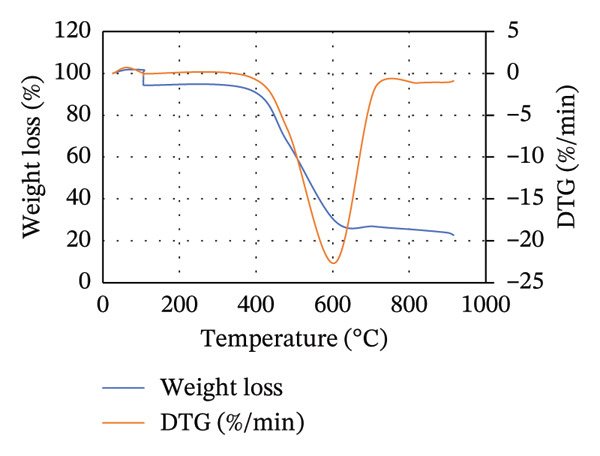
(e)

**Figure:**
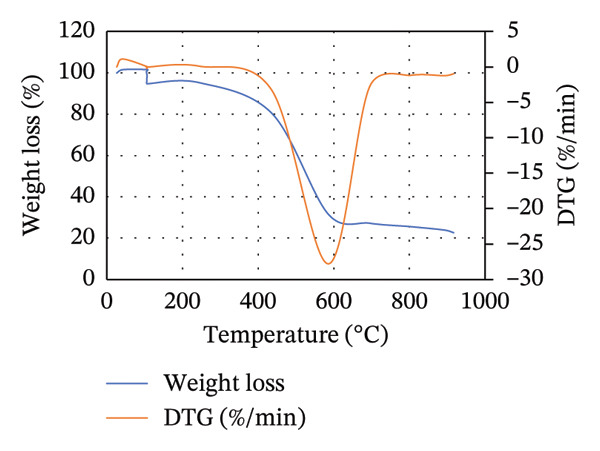
(f)

Figure 3 illustrates TGA and DTG curves for (a) 25MSW75CH, (b) 50MSW50CH, (c) 75MSW25CH, (d) 25MSW75CC, (e) 50MSW50CC, and (f) 75MSW25CC. For samples of 25MSW75CH, 50MSW50CH, and 75MSW25CH, moisture and light volatiles were lost between temperature ranges of 25°C–310°C, 25°C–340°C, and 25°C–370°C, respectively, as illustrated in Figures 3(a), 3(b), 3(c). Extensive thermal degradation occurred in ranges of 310°C–620°C, 340°C–630°C, and 370°C–650°C for similar ratios, respectively. Limited devolatilization in similar ratios occurred in temperature ranges of 620°C–915°C, 630°C–915°C, and 650°C–915°C, respectively.

On the other hand, for samples of 25MSW75CC, 50MSW50CC, and 75MSW25CC, moisture and light volatiles disposal occurred in ranges of 25°C–410°C, 25°C–420°C, and 25°C–430°C, respectively. Extensive thermal degradation occurred in ranges of 410°C–560°C, 420°C–605°C, and 430°C–580°C, while limited devolatilization occurred in ranges of 560°C–915°C, 605°C–915°C, and 580°C–915°C in ratios of 25:75, 50:50, and 75:25, respectively. This is illustrated in Figures 3(d), 3(e), 3(f). From Figures 3(a), 3(b), 3(c), 3(d), 3(e), 3(f), the thermal decomposition profiles of blended biomass samples are similar to those exhibited by the individual biomasses. This reveals that majority of the volatiles in the blended samples are liberated before biomass is heated to 500°C [32]. This illustrates that the properties of blended biomass can be comfortably upgraded through torrefaction.

DTG curves illustrate pronounced mass loss for all blends in temperature ranges of 280°C and 760°C. Zheng et al. [52] attribute such drastic mass losses to the release of volatile compounds possessed in the waste. Increasing the MSW composition in either the blend of MSW with CH or MSW with CC increases the rate of thermal degradation. This could be attributed to the higher reactivity and less stability of blend samples with high MSW compositions. It was also observed that the DTG curves for blended samples shifted towards higher temperatures compared to those of the individual biomass samples. This behavior is linked to improvement in thermal stability of the biochar [52].

## 4. Conclusion

Biomass, including MSW, CH, CC, and their blends, was characterized using proximate and ultimate analyses, lignocellulosic composition, calorific value, and thermal degradation behavior to assess their suitability for torrefaction‐based energy conversion. Among the individual feedstocks, MSW exhibited the highest moisture, volatile matter, and ash contents, while CH showed the highest fixed carbon content. Blending MSW with CH reduced its moisture, volatile matter, and ash contents while increasing fixed carbon, whereas blending MSW with CC mainly increased volatile matter and fixed carbon and reduced moisture and ash. From the ultimate analysis, MSW contained the highest hydrogen content, CH had the highest carbon content, and CC exhibited the highest oxygen, nitrogen, and sulfur contents. Blending MSW with CH enhanced carbon and nitrogen contents while reducing hydrogen, oxygen, and sulfur, whereas blending with CC increased carbon, oxygen, nitrogen, and sulfur while decreasing hydrogen. In terms of lignocellulosic composition, MSW had the lowest hemicellulose and cellulose contents, while CC had the lowest lignin content. Blending MSW with either CH or CC improved hemicellulose and cellulose contents but reduced lignin.

Although MSW alone exhibited the lowest calorific value, blending with CH or CC significantly enhanced its energy content. TGA revealed increased thermal degradation rates in blended samples, although excessive degradation was observed at high MSW proportions. Overall, blending MSW with agricultural residues improves its physicochemical and thermal properties, making it more suitable for torrefaction. Among the blends, 25MSW75CH demonstrated the lowest moisture and ash contents and the highest fixed carbon, carbon, lignin contents, and calorific value, identifying it as the most promising feedstock for torrefaction‐based WtE applications.

Limitations of this study include the absence of experimental torrefaction trials and gaseous or solid product yield analysis, as well as the restriction to laboratory‐scale physicochemical characterization without techno‐economic or life‐cycle assessment. In addition, only two agricultural residues were investigated, and the influence of torrefaction operating parameters (temperature, residence time, and heating rate) was not evaluated. Future work should therefore focus on performing torrefaction experiments on the identified optimal blend, assessing mass and energy yields, emissions, and fuel quality, and extending the analysis to pilot‐scale systems and economic feasibility studies to support industrial implementation.

## Author Contributions

Ibrahim Luqman Mpungu: conceptualization, investigation, methodology, data curation, writing–original draft preparation, and writing–review and editing.

Josphat Igadwa Mwasiagi: validation, writing–review and editing, and supervision.

Benson Dulo: methodology, data curation, writing–review and editing, and supervision.

Obadiah Maube: conceptualization, methodology, and supervision.

Patrick Nziu: conceptualization, validation, and supervision.

Ocident Bongomin: data curation, visualization, and writing–review and editing.

## Funding

This work was supported by the Africa Center of Excellence II in Phytochemical, Textile and Renewable Energy (ACE II‐PTRE).

## Conflicts of Interest

The authors declare no conflicts of interest.

## Data Availability

The data that support the findings of this study are available from the corresponding author upon reasonable request.
